# Light Gradient-Based Screening of *Arabidopsis thaliana* on a 384-Well Type Plant Array Chip

**DOI:** 10.3390/mi11020191

**Published:** 2020-02-12

**Authors:** Youn-Hee Park, Je-Kyun Park

**Affiliations:** 1 Department of Bio and Brain Engineering, Korea Advanced Institute of Science and Technology (KAIST), 291 Daehak-ro, Yuseong-gu, Daejeon 34141, Korea; younhee.park26@kaist.ac.kr; 2 KAIST Institute for Health Science and Technology, 291 Daehak-ro, Yuseong-gu, Daejeon 34141, Korea

**Keywords:** 384-well type plant array chip, *Arabidopsis thaliana*, germination test, light gradient module

## Abstract

*Arabidopsis thaliana* (*Arabidopsis*), as a model for plant research, is widely used for various aspects of plant science. To provide a more sophisticated and microscopic environment for the germination and growth of *Arabidopsis*, we report a 384-well type plant array chip in which each *Arabidopsis* seed is independently seeded in a solid medium. The plant array chip is made of a poly(methyl methacrylate) (PMMA) acrylic material and is assembled with a home-made light gradient module to investigate the light effects that significantly affect the germination and growth of *Arabidopsis*. The light gradient module was used to observe the growth pattern of seedlings according to the intensity of the white light and to efficiently screen for the influence of the white light. To investigate the response to red light (600 nm), which stimulates seed germination, the light gradient module was also applied to the germination test. As a result, the germination results showed that the plant array chip can be used to simultaneously screen wild type seeds and phytochrome B mutant seeds on a single array chip according to the eight red light intensities.

## 1. Introduction

The genetic diversity and viability of plants depend on the environmental factors that are applied to solid media, as well as the intrinsic factors of the seed. Because of the effects on complex environmental stimuli such as temperature, light, humidity, pressure, pH, and microorganisms [1], finding an optimal environment for plant seeding and culture has been recognized as one of the most important issues in the field of plant science. To date, *Arabidopsis thaliana* (*Arabidopsis*), which is a well-known model plant, has been used for the study of plant science and genetic engineering [2]. However, the seeds of *Arabidopsis*, typically up to several hundred micrometers in size, are difficult to handle by hand. Conventional seeding in a Petri dish also requires the continuous tracking of each object to observe its temporal variation, but it is difficult to observe the size and shape of the seeds because there are no physical partitions in an agarose growth environment [3]. Accordingly, as the number of seeds that are associated with several environmental factors increases, a more effective monitoring system is required to simultaneously observe the germination and growth rate of the plant.

Factors that affect the growth of naturally grown plants can be classified into two broad categories. The first is external factors such as temperature [4], light [5] soil water treatment [6], and pollutants [7]. The second is internal factors [8], including nutrients, genetic factors, and growth regulators. In particular, light is one of the most important factors for the survival of plants and has a direct influence on growth and development [9]. The plant contains blue light receptors [10] and red light receptors [11] in its light-sensing system. Phytochrome, a red light receptor, plays an important role in the germination of plants [12], and it is activated by 660 nm wavelength red light. When phytochrome is activated, the germination mechanism inside the seed is activated to create an internal condition that can start germination. 

Microfluidics has been applied to study the handling of *Arabidopsis* seeds for screening purposes [13,14,15]. By using microfluidic technology, small-sized *Arabidopsis* seeds can be easily trapped in each chamber one by one, effectively managing many seeds simultaneously. Additionally, a liquid medium, which is very easy to change or deliver into the chamber, has been used to provide all nutrients for growth. A fully automated miniature culture chamber that enables a supply of liquid medium and real-time environmental control has also been developed [16]. However, due to this culture chamber’s low versatility, the observation of a specific root phenotype is still limited [17,18,19,20,21,22,23,24]. Previously, we developed a poly(dimethylsiloxane) (PDMS)-based plant array chip for the germination and growth screening of plants under a variety of conditions [25]. Because the absorption and adsorption of hydrophobic small molecules is an intrinsic property of PDMS, there is a limitation to maintain each PDMS-based well in a completely independent environment, depending on the characteristics of the molecules within an agar medium. As an alternative, poly(methyl methacrylate) (PMMA) is suitable to overcome this limitation. A PMMA plate can be fabricated by laser machining, which is a more efficient fabrication method than soft lithography with PDMS. In addition, since the previous chip design had a linear channel shape, twenty seedlings in the same channel could affect each other. The volume of the agarose medium for one seed was also small compared to that of the conventional Petri dish, which resulted in the effective concentration of the molecules contained in the agarose medium being insufficient for the germination and growth of the seed. To clearly distinguish the variation between the seedlings, the cultivation must take place in an independent container. 

In this study, we report a 384-well type plant array chip that is suitable for setting laboratory environmental conditions. The plant array chip is made of a PMMA acrylic material and is assembled with a light gradient module to investigate light effects that significantly affect the germination and growth of *Arabidopsis*. If the seeds germinated by red light are exposed to darkness, the seedlings are etiolated, in which the elongation of hypocotyl is dominant, and the etiolated seedlings are yellowish-white due to a lack of chlorophyll [26]. Because of this mechanism, the ratio of red light to far-red light plays a very important role in seed germination [27]. For this reason, red or far-red light-emitting diodes (LEDs) were used independently or independently with the light of two wavelengths for the phytochrome study [28]. Accordingly, the ratio of light or the intensity of light is a very important experimental factor. In this study, a light gradient module was exploited to create various light intensity conditions in a 384-well type plant array on a single chip, which can be used for screening of germination and growth by light. By using a light gradient module, we demonstrate a highly efficient tool for changing germination conditions and controlling the germination process in a plant array chip.

## 2. Materials and Methods

### 2.1. Design and Concept

**Figure 1:**
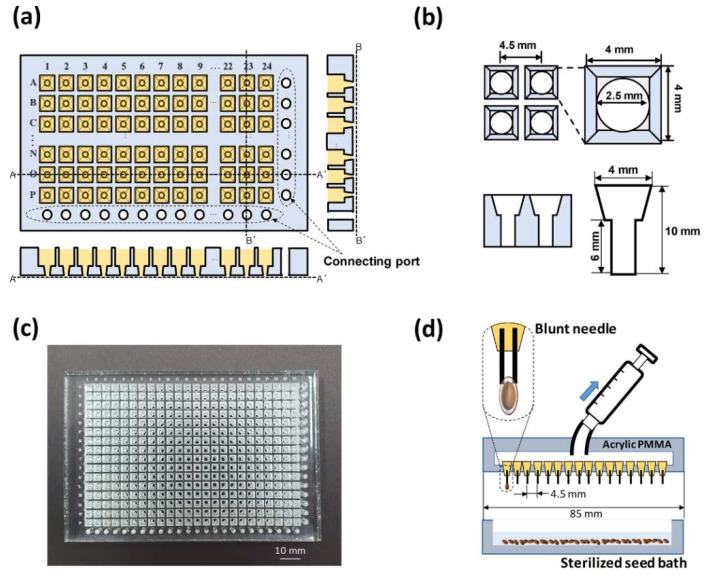
Configuration of a 384-well type plant array chip that was fabricated by laser machining with an acrylic poly(methyl methacrylate) (PMMA) plate. (**a**) Schematic top and side views of a 384-well type plant array chip. (**b**) The dimensions of each well that was fabricated in a 384-well type plant array chip. (**c**) Photograph of the fabricated plant array chip compatible with a conventional well plate (127 × 85 × 10 mm). (**d**) A home-made micro-vacuum seeder for a high efficiency of seeding on a 384-well type plant array chip. There were 16 needles in the seeder head.

To develop a plant culture platform that can apply various media conditions to one chip for highly efficient screening, a partitioning section for multiple seeding is required on a single plant chip. By integrating multiple channels as a fluidic module into a plant chip, each seed could be screened without affecting any others. First, we designed a 384-well type plant array chip (127 × 85 × 10 mm) with the same dimensions (24 columns × 16 rows) as a conventional, standardized multi-well plate, which was engraved with numeric digits (1–24) and alphabetic letters (A–P) for each column and row, respectively (Figure 1a). The plant array chip had two functional structures that included 384 well arrays with a hole in the bottom in each well and 40 connecting ports. The connecting ports served to supply 24 wells in the row direction and 16 wells in the column direction with nutrient- or substance-containing liquids, thereby enabling a variety of microfluidic modules to be attached to the bottom of the plant array chip. In addition, the cross-section of the single well became narrower through the *z*-axis from the top. This shape prevented the agarose block from exiting from a single well (Figure 1b). 

### 2.2. Fabrication of a 384-Well Type Plant Array Chip

A 384-well type plant array chip was fabricated by using an acrylic PMMA plate, which was processed by both laser engraving and laser cutting on the surface of a PMMA plate with a thickness of 1 cm (Figure 1c). The plant array chip was filled with an agarose medium precursor. A high-temperature sterilized medium was poured into a container (about 70 mL), and the plant array chip was immersed in the container. When each well was filled with liquid agarose, the plant array chip remained in a clean bench until the agarose solidifies. The detailed fabrication procedures of a 384-well type plant array chip and agarose solidification are shown in Supporting Figure S1. Prior to conducting all experiments, the fabricated array chip was soaked in 70% ethanol for 1 h to sterilize it. 

To facilitate the multiple seeding process on the 384-well structure, a homemade micro-vacuum seeder, in which a needle array consisting of 16 needles (27 G, I.D. 210 μm) was used to catch each seed one by one, was exploited to plant the 384 seeds (Figure 1d; Video S1). The head of the seeder was fabricated with an acrylic PMMA plate by laser machining. Consequently, the sterilized seeds (with an average diameter of 500 μm) were transferred to a seed bath that was filled with distilled water. It was possible to capture 16 seeds at the same time. In this way, the captured seeds were immediately transferred to the plant array chip. Accordingly, the seeding time was reduced from 40 to 5 min compared to the conventional manual process.

### 2.3. Integration of a Light Gradient Module

**Figure 2:**
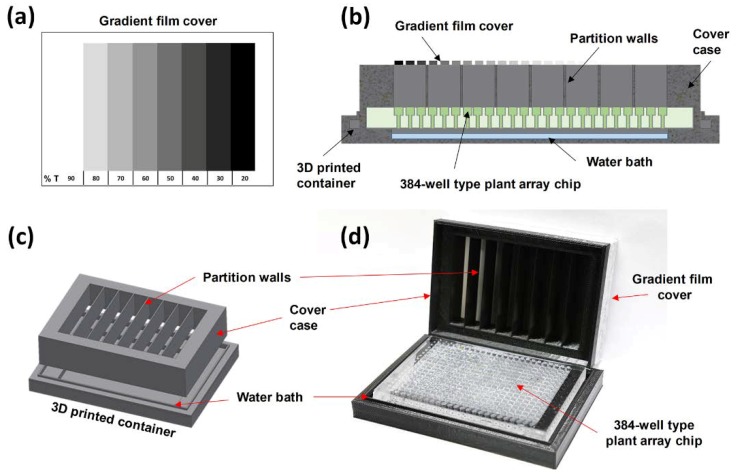
A light gradient module and the integration of a plant array chip. (**a**) Schematic of a laser-printed light gradient film with eight different light transmittances. % T indicates the transmittance expressed as a fraction of 100. (**b**) Assembly of a 384-well type plant array chip in a light gradient module. (**c**) Schematic of a 3D printed light gradient module case. (**d**) Photograph of a light gradient module and a 384-well type plant array chip.

In order to observe the effect of the light intensity difference on a single chip, only the light in the vertical direction should affect the seeds. In addition, in order to investigate the desired light intensity in each well, the light in the other wells should not be affected by each other. For this purpose, we designed a light gradient module that consisted of a cover case with a gradient film that was attached to the partition walls and a water bath plate. By using the light gradient module, the plant array chip was divided into eight compartments, and the 48 (=16 × 3) seeds that were contained in each compartment were exposed to the same intensity. To control the transmittance of light, a gradient film was designed to have eight transmissivities on a transparent film (Figure 2a). Because all control experiments were carried out within the transparent plastic Petri dish, the effective light intensity of the modules was reduced compared to control groups. When the transmittance of the clear region (non-achromatic-dye region) of the overhead projector (OHP) film was measured, it was confirmed that the intensity decreased by 10%. To express this situation, the maximum transmittance of the module was assumed to be 90%. By adjusting an RGB (red, green, and blue) color value on an OHP film by using a laser printer, achromatic dyes of different brightness levels were deposited on a transparent film. The RGB color value indicated the intensity in red, green, and blue intensity, with each color intensity having integer values from 0 to 255. The printed film then adhered to the printed side down on the cover case (Figure 2b). The cover case was fabricated with a fused deposition modeling (FDM) 3D printer (3DISON Multi; Rokit, Seoul, Korea) by using a black colored polylactic acid filament (Figure 2b,c). Finally, the printed film was attached on the top surface of the cover case to provide eight compartments, and the bottom of each partition wall was completely in contact with the plant array chip so that no interference occurred. The detailed assembly procedure of the light gradient module with the plant array chip is shown in Supporting Figure S2.

### 2.4. Incubation of Arabidopsis in a 384-Well Type Plant Array Chip

*Arabidopsis* was kindly obtained from Professor Giltsu Choi at the Department of Biological Sciences, KAIST. All plants were the Columbia-0 (Col-0) background. The water bath plate was filled with 10 mL of distilled water, and then a fully seeded array chip was put on the water bath plate. The plant array chip was then covered with a light gradient module. The light gradient module that contained the array chip was sealed with a micropore paper tape at the joint. An incubator (MI-20A; S.N.T. Co., Ltd, Jinju, Korea) was used to adjust the temperature of the environment and the wavelength of the light source. The humidity inside the incubator was maintained at 70% to 80%, and an on/off system of the red LEDs was equipped inside the incubator. After the cold treatment at 4 °C for 48 h (in the dark), the plant array chip was immediately exposed to red light in an incubator at 23 °C for 5 min. Then, it was kept in the dark for 66 or 72 h, depending on the experimental condition, to maintain the darkness state. To observe the growth by white light intensity, the assembled plant array chip, after 5 min exposure to the same red light followed by the cold treatment, was transferred to an incubator at 23 °C for 72 h in the dark before being exposed to white light.

The germination rate indicates the ratio of the germinated seed number within a certain period as compared to the number of seeds in the plant array chip. The seed germination on the plant array chip was observed by measuring the radical length by using an image processing program (ImageJ, https://imagej.nih.gov/ij/) based on the photograph by a stereomicroscope (Nikon; Japan) [25]. 

## 3. Results and Discussion

### 3.1. Germination and Growth Assay on a 384-Well Type Plant Array Chip

**Figure 3:**
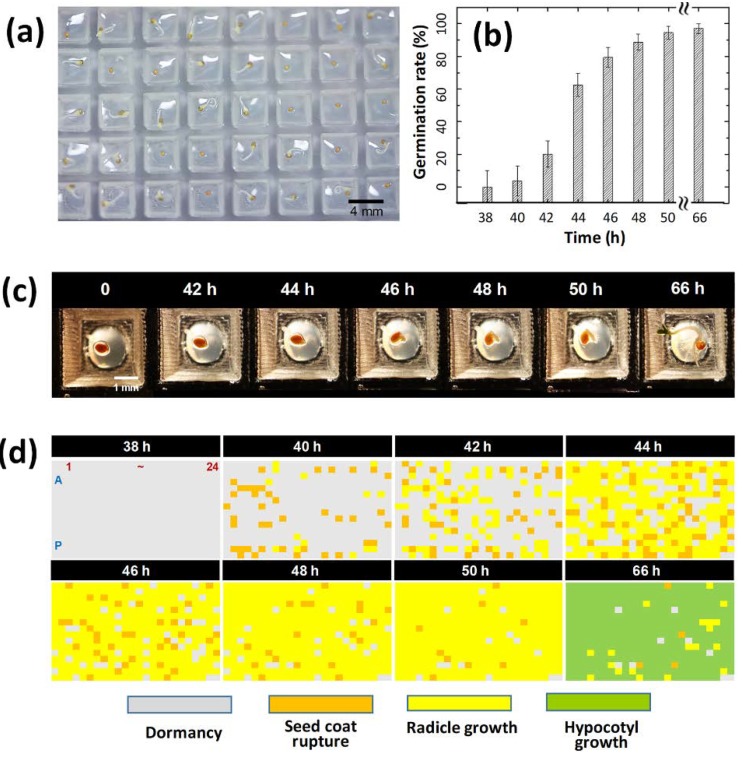
Demonstration of cultivation on the 1/25 Murashige and Skoog (MS) medium-conditioned plant array chip. (**a**) Observation photograph of seedlings on the plant array chip from above. (**b**) Graph of germination rate versus time on a 384-well type plant array chip. (**c**) Stereomicroscopic images of a single well, depending on the time. The germination of the seed began after 42 h. The seedling was observed after 66 h. (**d**) Tracking map results according to the four germination stages, including dormancy, seed coat rupture, radicle growth, and hypocotyl growth, all of which were obtained from the top-view photograph of the whole array chip.

To demonstrate a 384-well type plant array chip, the germination and growth of the seed were observed at a 1/25 Murashige and Skoog (MS) media condition. As shown in Figure 3a, the seeds could be observed without interfering because of the transparent acrylic PMMA plate. Compared to the previous study, interferences between seedlings were reduced because the dimensions of the well were larger than the existing array chip, with an area assigned to a single seedling [25]. By a simple demonstration, we confirmed that the basic environmental function was well-controlled on the 384-plant array chip. The germination rate was plotted over time (Figure 3b). It was confirmed that the germination rate increased with time, and after about 46 h, the germination rate increased to 80% or more, showing the same tendency as the Petri dish protocol.

When observing the germination of 384 *Arabidopsis* seeds by using a 384-array chip, the seeds were divided into individual wells so that the seed status could be tracked over time (Figure 3c). To screen all differences between objects for the 384 seeds that were planted on the plant array chip, a color mapping result of the plant array chip according to the time was created (Figure 3d). In this study, we manually scored the stages of the seeds. The seed germination process was divided into four stages: dormancy (gray), seed coat rupture (orange), radicle growth (yellow), and hypocotyl growth (green). In this way, the color map showed not only the germination rate but also the germination stage and the growth rate of each seed. Accordingly, the color map at 66 h made it possible to distinguish between a seed that stopped germination in the seed coat rupture stage (orange) and a seed that did not germinate at all, so that the exact nature of each object could be determined.

### 3.2. Effect of White Light Intensity on Arabidopsis Growth

**Figure 4:**
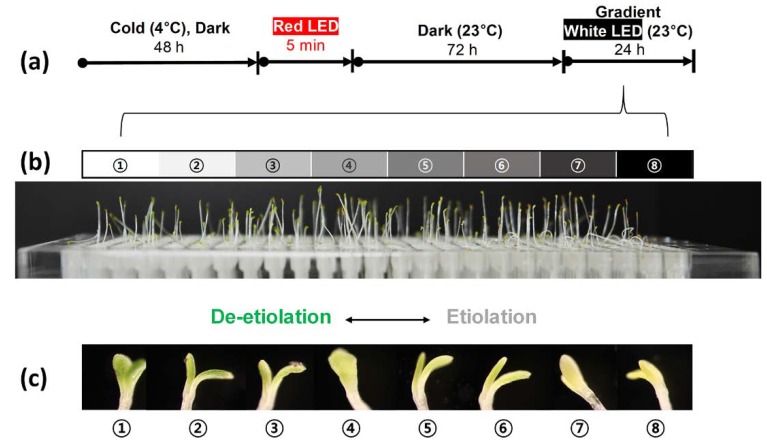
Effect of the white light on the etiolation, as observed with a light gradient module. (**a**) Before exposure to a white light gradient, Arabidopsis was kept at 4 °C for 48 h, exposed to red light in an incubator at 23 °C for 5 min, and then darkened for 72 h. (**b**) Eight sections (①–⑧) of the plant array chip were exposed to different white light intensities by using a light gradient film. (**c**) Photographs of the seedling that were obtained at different sections on the plant array chip.

In this study, a white light gradient was used to observe the effect of light on the growth of *Arabidopsis* after germination (Figure 4a). At first, the germinated seeds were continuously exposed to the dark environment for 72 h to induce etiolation [29]. After that, the seedlings were exposed to white light for about 24 h to induce de-etiolation, and the change in morphology was observed [30]. Figure 4b shows the result after inducing the de-etiolation of a seedling from sections ① to ⑧ of the light gradient film with different light transmittances. The seedling in the figure showed that the vertical growth of the plants was due to negative gravitropism, which is known as a typical growth phenomenon in the opposite direction of gravity and is exaggerated in etiolated plants. Since the etiolation occurred at the same time, the total length of the seedling was almost the same. When the etiolation was induced, the morphology of the seedling was not as green as the cotyledon of the seedling, as shown in Figure 4c⑧. However, when the light was irradiated for 24 h, it was confirmed that cotyledon greening occurred according to the light intensity (Figure 4c). The opening phase of cotyledon also differed depending on the intensity of the light. The lower the light intensity, the less frequently cotyledon opening occurred. Based on the rate of de-etiolation of the seedling according to the transmittance, we confirmed that the light gradient module was working properly.

### 3.3. Effect of Red Light Intensity on Germination Rate

**Figure 5:**
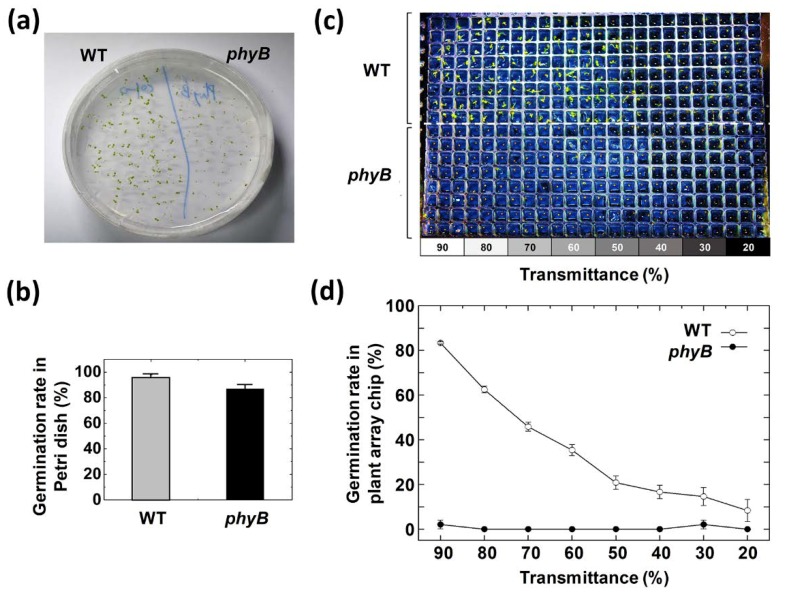
Effect of the red light intensity on the germination rate of wild type (WT) and phytochrome B mutant (*phyB*) seeds. (**a**,**b**) Germination test results after 72 h of incubation under a red light condition of 2 μmol·m^−2^·s^−1^ in a Petri dish. (**c**,**d**) Germination comparison of WT and *phyB* seeds in a plant array chip. (**c**) Top view of a single plant array chip showing different germination behaviors. (**d**) Graph of the germination rates in WT and *phyB* seeds depending on the red light gradient on the plant array chip.

Red light directly affects the time at which *Arabidopsis* starts germination. In particular, red light acts on phytochrome to stimulate the germination of *Arabidopsis* seeds so that the germination rate is dependent on the intensity of the red light. Therefore, the optimal red light intensity with the highest germination rate is important [31]. In this study, two types of seeds, wild type (WT) and phytochrome B mutant (*phyB*), were screened on a 384-well type plant array chip for the germination rate according to eight red light intensities in one array chip by using a light gradient module. Theoretically, the germinations of WT seeds are induced by red light, and their germination rates are proportional to the red light’s intensity. On the other hand, *phyB* seeds are known to be less sensitive to red light than WT seeds [32]. Therefore, the effects of red light on the germination of seeds can be easily screened by using the light gradient module. To check the viability of WT seeds and *phyB* seeds, the germination test was carried out by using a Petri dish under the same medium conditions. It was found that WT seeds and *phyB* seeds in the Petri dish had a germination rate of approximately 95% and 87%, respectively, under a red light condition of 2 μmol·m^−2^·s^−1^ (Figure 5a,b).

To compare the germination rates of two different seeds in the eight compartments of a 384-well type plant array chip, WT seeds were planted in rows A to H, while *phyB* seeds were planted in rows I to P in the plant array chip. After the cold treatment at 4 °C for 48 h in the darkness, the light gradient module-assembled array chip was exposed to red light for 5 min. Germination rates were measured according to the transmittance of the red light (Figure 5c,d) after 72 h of incubation at 23 °C in the darkness.

As a result, the germination rate of the WT seeds was decreased as the transmittance of red light was decreased. Though the same single chip was used, *phyB* seeds did not germinate at any of the ranges of transmittance. The results showed that the germination response of the *phyB* seeds by the red light was lower than that of the WT seeds. Compared to the test results in the Petri dish, the germination rates were different. One of the possible reasons for this was that the red light transmittance of the gradient film was lower than that of the transparent plastic Petri dish. For an accurate screening of a *phyB* seed by using the light gradient module, a film with high transmittance must be used, or the power of the red light source in a chamber must be higher than 2 μmol·m^−2^·s^−1^.

## 4. Conclusions

A 384-well type plant array chip was fabricated by processing a PMMA plate with laser machining. The plant array chip could easily load the agar medium into 384 wells, which were seeded with 384 seeds into each well. Since each well represented a completely independent environment, the growth environment of each seed could be more strictly limited. A germination test that was performed on a 384-array chip showed that the array chip can be a suitable platform for screening the germination and growth of *Arabidopsis*. It was also successfully demonstrated that a light gradient module that was developed for the 384-array chip observed the effect of light more precisely by blocking all other light except vertical light. By using this module, we were able to investigate the effects of the intensity of the red and white light that promote germination and growth, respectively.

Meanwhile, a highly efficient germination screening platform can be applied for the screening of repetitive conditions. For example, in the early stages of herbicide development or seed development, numerous germination rate tests have to be repeated for specific chemicals. This platform helps in the identification of side effects on a photoreceptor and its metabolism, which are essential for the survival of plants, especially in genetically modified seeds. For practical applications of plant array chips, nutrients or medium solutions should be continuously supplied to each well of the plant array chip. In this sense, our chip design is convenient for interfacing with a microfluidic module that efficiently performs complex functions through dynamic external stimuli to supply additional media into each well of the plant array chip.

However, there are several limitations. Because the PMMA-based plant array chip is transparent, interference light affects each well. Therefore, it is not possible to fully control the light that affects the lower hypocotyl and root in current array chips. In addition, due to the small size of the well (4 mm), only germination and early-stage growth can be screened. Our plant array chip could be implemented with wells of different sizes to allow for long-term observation.

## Supplementary Materials

The following are available online at https://www.mdpi.com/2072-666X/11/2/191/s1. Figure S1. Fabrication process of a 384-well type array chip by using an acrylic PMMA plate, Figure S2. Assembly protocol of a light gradient module and a 384-well type plant array chip, Video S1. Operation of micro-vacuum seeder.

## References

[1] Houle D., Govindaraju D.R., Omholt S. (2010). Phenomics: The next challenge. Nat. Rev. Genet..

[2] Bowman J. (1994). Arabidopsis: An Atlas of Morphology and Development.

[3] Iyer-Pascuzzi A.S., Symonova O., Mileyko Y., Hao Y., Belcher H., Harer J., Weitz J.S., Benfey P.N. (2010). Imaging and analysis platform for automatic phenotyping and trait ranking of plant root systems. Plant Physiol..

[4] Stavang J.A., Gallego-Bartolomé J., Gómez M.D., Yoshida S., Asami T., Olsen J.E., García-Martínez J.L., Alabadí D., Blázquez M.A. (2009). Hormonal regulation of temperature-induced growth in Arabidopsis. Plant J..

[5] Liu X., Qin T., Ma Q., Sun J., Liu Z., Yuan M., Mao T. (2013). Light-regulated hypocotyl elongation involves proteasome-dependent degradation of the microtubule regulatory protein WDL3 in *Arabidopsis*. Plant Cell.

[6] Zhang Y., Kendy E., Qiang Y., Changming L., Yanjun S., Hongyong S. (2004). Effect of soil water deficit on evapotranspiration, crop yield, and water use efficiency in the North China Plain. Agric. Water Manag..

[7] Koziol M.J., Whatley F.R. (1984). Gaseous Air Pollutants and Plant Metabolism.

[8] Chailakhyan M.K. (1968). Internal factors of plant flowering. Ann. Rev. Plant Physiol..

[9] Kami C., Lorrain S., Hornitschek P., Fankhauser C. (2010). Chapter two-light-regulated plant growth and development. Curr. Top. Dev. Biol..

[10] Kasahara M., Swartz T.E., Olney M.A., Onodera A., Mochizuki N., Fukuzawa H., Asamizu E., Tabata S., Kanegae H., Takano M. (2002). Photochemical properties of the flavin mononucleotide-binding domains of the phototropins from Arabidopsis, Rice, and *Chlamydomonas reinhardtii*. Plant Physiol..

[11] Li J., Li G., Wang H., Deng X.W. (2011). Phytochrome signaling mechanisms. Arab. B.

[12] Shinomura T., Nagatanit A., Hanzawa H., Kubotat M., Watanabet M., Furuya M. (1996). Action spectra for phytochrome A- and B-specific photoinduction of seed germination in *Arabidopsis thaliana*. Proc. Natl. Acad. Sci. USA.

[13] Tatic-Lucic S. (2007). Applications of bioMEMS in cell-related research. Semi. Device Research Symp..

[14] Takayama S., Ostuni E., LeDuc P., Naruse K., Ingber D.E., Whitesides G.M. (2001). Subcellular positioning of small molecules. Nature.

[15] Horade M., Yanagisawa N., Mizuta Y., Higashiyama T., Arata H. (2014). Growth assay of individual pollen tubes arrayed by microchannel device. Microelec. Eng..

[16] Jiang H., Wang X., Aluru M.R., Dong L. (2019). Plant miniature greenhouse. Sens. Actuator A Phys..

[17] Jiang H., Xu Z., Aluru M.R., Dong L. (2014). Plant chip for high-throughput phenotyping of *Arabidopsis*. Lab Chip.

[18] Agudelo C.G., Nezhad A.S., Ghanbari M., Naghavi M., Packirisamy M., Geitmann A. (2013). TipChip: A modular, MEMS-based platform for experimentation and phenotyping of tip-growing cells. Plant J..

[19] Grossmann G., Guo W., Ehrhardt D.W., Frommer W.B., Sit R.V., Quake S.R., Meier M. (2011). The RootChip: An integrated microfluidic chip for plant science. Plant Cell.

[20] Jiang H., Jiao Y., Maneesha M.R., Dong L. (2012). Electrospun nanofibrous membranes for temperature regulation of microfluidic seed growth chips. J. Nanosci. Nanotechnol..

[21] Meier M., Lucchetta E.M., Ismagilov R.F. (2010). Chemical stimulation of the *Arabidopsis thaliana* root using multi-laminar flow on a microfluidic chip. Lab Chip.

[22] Busch W., Brad T.M., Bradley M., Mace D.L., Twigg R.W., Jung J., Pruteanu-Malinici I., Kennedy J.S., Fricke G.K., Clark R.L. (2012). A microfluidic device and computational platform for high-throughput live imaging of gene expression. Nat. Methods.

[23] Sanati Nezhad A. (2014). Microfluidic platforms for plant cells studies. Lab Chip.

[24] Parashar A., Pandey S. (2011). Plant-in-chip: Microfluidic system for studying root growth and pathogenic interactions in *Arabidopsis*. Appl. Phys. Lett..

[25] Park Y.-H., Lee N., Choi G., Park J.-K. (2017). Plant array chip for the germination and growth screening of *Arabidopsis thaliana*. Lab Chip.

[26] Chory J., Peto C.A., Ashbaugh M., Saganich R., Pratt L., Ausubel F. (1989). Different roles for phytochrome in etiolated and green plants deduced from characterization of *Arabidopsis thaliana* mutants. Plant Cell.

[27] Jankowska-blaszczuk M., Daws M.I. (2007). Impact of red: Far red ratios on germination of temperate forest herbs in relation to shade tolerance, seed mass and persistence in the soil. Funct. Ecol..

[28] Halliday K.J., Koornneef M., Whitelam G.C. (1994). Phytochromes B, D, and E act redundantly to control multiple physiological responses in Arabidopsis. Plant Physiol..

[29] Jensen P.J., Hangarter R.P., Estelle M. (1998). Auxin transport is required for hypocotyl elongation in light-grown but not dark-grown Arabidopsis. Plant Physiol..

[30] Beligni M.V., Lamattina L. (2000). Nitric oxide stimulates seed germination and de-etiolation, and inhibits hypocotyl elongation, three light-inducible responses in plants. Planta.

[31] Reed J.W., Nagpal P., Poole D.S., Furuya M., Chory J. (1993). Mutations in the gene for the red/far-red light receptor phytochrome B alter cell elongation and physiological responses throughout Arabidopsis development. Plant Cell.

[32] Leyser O., Day S. (2009). Mechanisms in Plant Development.

