# Supplementary material for: Light Gradient-Based Screening of Arabidopsis thaliana on a 384-Well Type Plant Array Chip
Source: Micromachines (Basel). 2020 Feb 12;11(2):191. doi: 10.3390/mi11020191 (PMC7074641; doi:10.3390/mi11020191)
Supplement: Supplementary file 1 [file micromachines-11-00191-s001.zip › micromachines-691189-suppl._corrections_clean.docx]

Supplementary Information: Light Gradient-Based Screening of *Arabidopsis thaliana* on a 384-Well Type Plant Array Chip

Youn-Hee Park^1^ and Je-Kyun Park ^1,2,^*

^1^ Department of Bio and Brain Engineering, Korea Advanced Institute of Science and Technology (KAIST), 291 Daehak-ro, Yuseong-gu, Daejeon 34141, Republic of Korea; younhee.park26@kaist.ac.kr

^2^ KAIST Institute for Health Science and Technology, 291 Daehak-ro, Yuseong-gu, Daejeon 34141, Republic of Korea

***** Correspondence: jekyun@kaist.ac.kr; Tel.: +82-42-350-4315


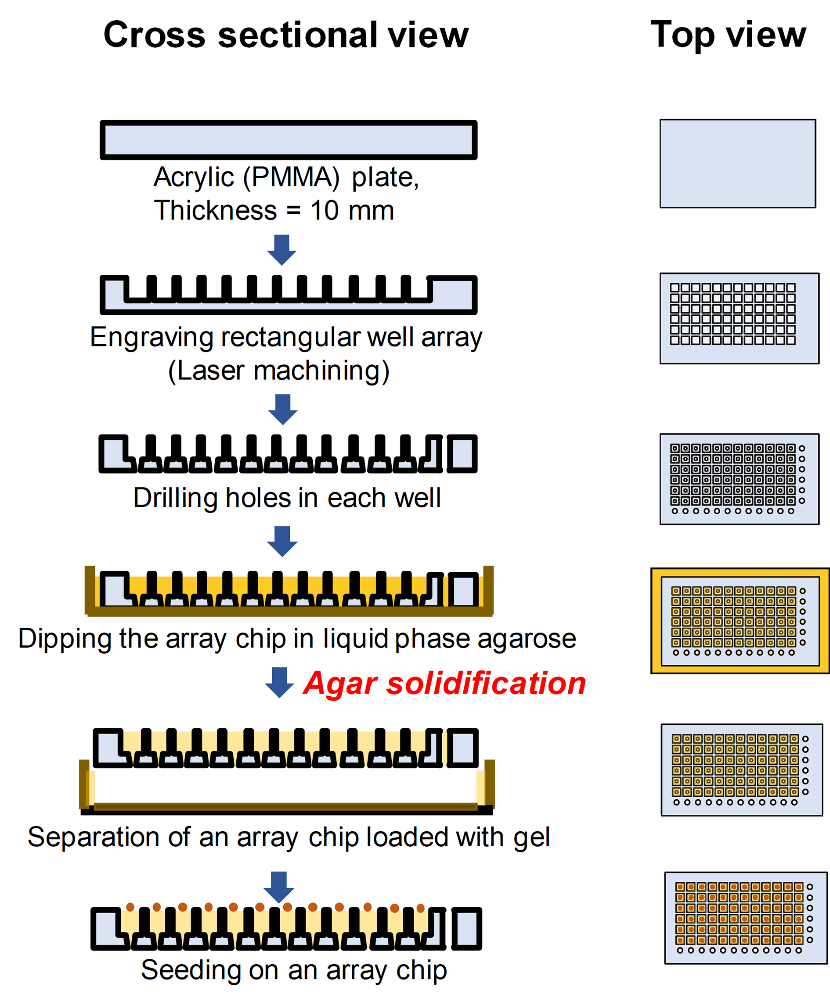


**Figure S1.** Fabrication process of a 384-well type array chip by using an acrylic PMMA plate.


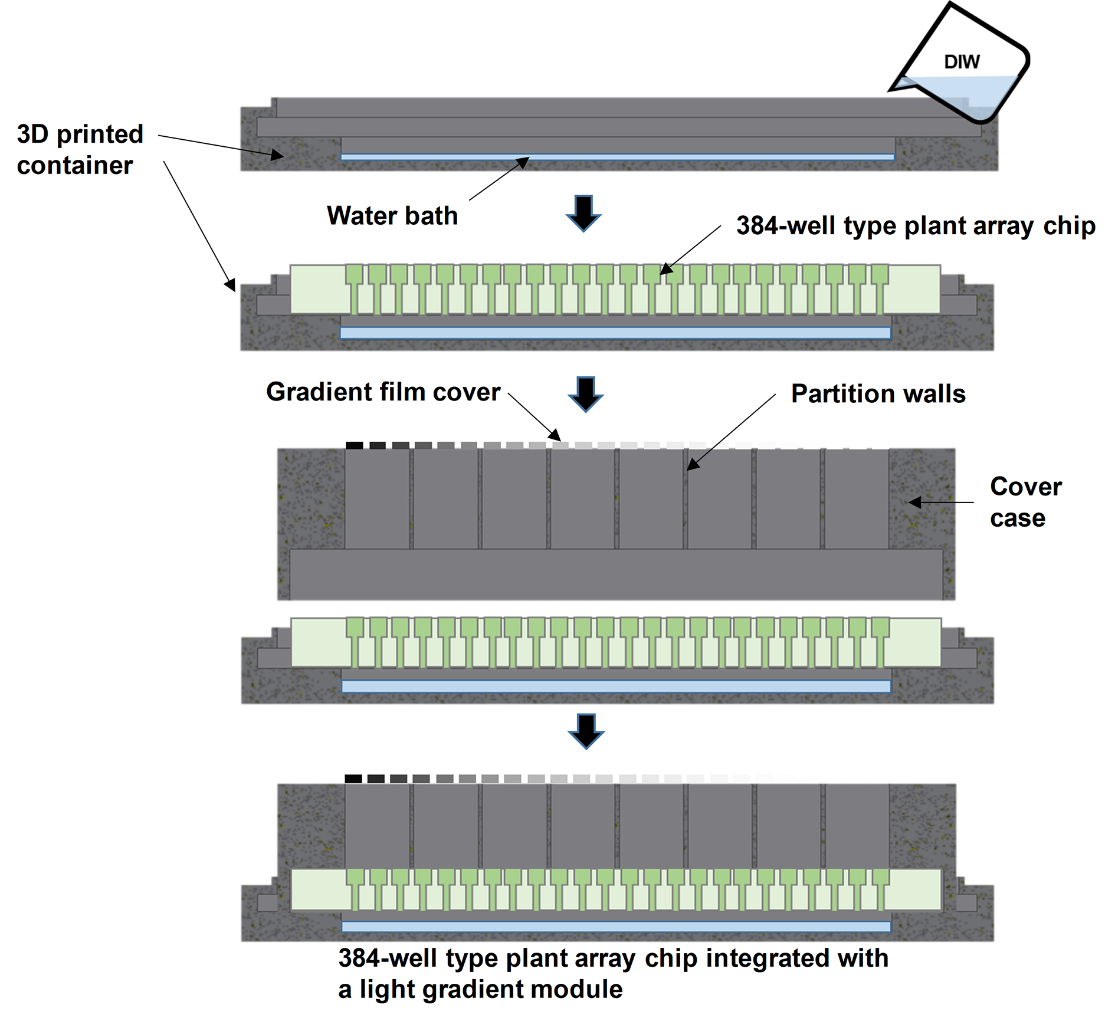


**Figure S2.** Assembly protocol of a light gradient module and a 384-well type plant array chip.
